# Outcomes of TB Treatment by HIV Status in National Recording Systems in Brazil, 2003–2008

**DOI:** 10.1371/journal.pone.0033129

**Published:** 2012-03-21

**Authors:** Mauro Sanchez, Patricia Bartholomay, Denise Arakaki-Sanchez, Donald Enarson, Karen Bissell, Draurio Barreira, Anthony Harries, Afrânio Kritski

**Affiliations:** 1 Department of Public Health, University of Brasilia, Brasilia, Brazil; 2 Strategic Information Unit, National TB Control Program, Brazilian Ministry of Health, Brasilia, Brazil; 3 Department of Research, International Union Against Tuberculosis and Lung Disease, Paris, France; 4 Academic Tuberculosis Program, Federal University of Rio de Janeiro, Rio de Janeiro, Brazil; National Institute for Infectious Diseases (L. Spallanzani), Italy

## Abstract

**Background:**

Although the Brazilian national reporting system for tuberculosis cases (SINAN) has enormous potential to generate data for policy makers, formal assessments of treatment outcomes and other aspects of TB morbidity and mortality are not produced with enough depth and rigor. In particular, the effect of HIV status on these outcomes has not been fully explored, partly due to incomplete recording in the national database.

**Methodology/Principal Findings:**

In a retrospective cohort study, we assessed TB treatment outcomes, including rates of cure, default, mortality, transfer and multidrug resistant TB (MDR-TB) among a purposively chosen sample of 161,481 new cases reported in SINAN between 2003 and 2008. The study population included all new cases reported in the six States with the highest level of completeness of the HIV status field in the system. These cases were mostly male (67%), white (62%), had pulmonary TB (79%) and a suspect chest X ray (83%). Treatment outcomes were best for those HIV negative cases and worst for those known HIV positive patients (cure rate of 85.7% and 55.7% respectively). In multivariate modeling, the risk of having an unfavorable outcome (all outcomes except cure) was 3.09 times higher for those HIV positive compared with those HIV negative (95% CI 3.02–3.16). The risk of death and default also increased with HIV positivity. The group without a known HIV status showed intermediate outcomes between the groups above, suggesting that this group includes some with HIV infection.

**Conclusions:**

HIV status played an important role in TB treatment outcomes in the study period. The outcomes observed in those with known HIV were poor and need to be improved. Those in the group with unknown HIV status indicate the need for wider HIV testing among new TB cases.

## Introduction

Tuberculosis and HIV represent major public health challenges [1], although effective case management is currently available for those who are detected. Integration of TB and HIV/AIDS programs is needed in countries where this dual epidemic occurs, but in practice few countries have implemented such coordinated activities [2].

Brazil ranks as the 19^th^ country in the list of 22 high burden countries (HBC) for tuberculosis, with an estimated number of cases per year of around 71,000, with 4,800 deaths in TB patients each year. In Brazil, TB is the third cause of death by infectious diseases and the leading cause of death among AIDS patients [3]. The HIV epidemic in Brazil has stabilized in recent years, with the prevalence of HIV among adults (15 to 49 years old) remaining at about 0.6% between 2000 and 2006. [4] Official figures from the Ministry of Health indicate that in 2006 the estimated number of people living with HIV/AIDS (PLWHA) was approximately 630,000 [4].

The World Health Organization (WHO) commented that TB HIV collaborative activities are still not fully implemented in Brazil, in spite of localized initiatives [2]. Although the Brazilian program for HIV/AIDS was recognized internationally and tuberculosis has been declared a priority by the Brazilian government in 2003, it is observed that both programs' recommendations were focused on therapeutic regimens addressing the issue of interaction between rifampicin and antiretroviral drugs. In both programs there were no clear guidelines on how to reduce the burden of TB in PLWHA and vice versa. To illustrate this delay in the implementation of TB/HIV collaborative activities in the country, we can highlight the fact that in 2008, out of the 12 indicators of TB/HIV collaborative activities recommended by the WHO [5], only three were available using routinely collected information. The National TB Control Program is currently undertaking a large scale project in the Southern region of Brazil to foster these activities and demonstrate the impact that a well-organized and coordinated response to the dual epidemic could have.

There is a strong link between TB and HIV, in that HIV increases the likelihood of developing TB [6] and, if untreated, is more likely to be associated with death while on TB treatment [7]. Moreover, the detection of HIV in TB patients enables access to HIV care and treatment.

Furthermore, HIV influences the duration of infectiousness of TB [8]. HIV-infected individuals may have a shorter infectious period due to a more rapid progression to TB disease. Alternatively, due to a lack of timely diagnosis, they may stay infectious for longer than an individual without HIV. Therefore, TB infectiousness is related to access to care and timely diagnosis of TB in PLWHA [8].

There are scarce data from large cohorts of patients entered into the routine national reporting systems on the effect of HIV status on TB treatment outcomes. We therefore conducted a study to determine the TB treatment outcomes by category of HIV serostatus of a cohort of all new cases of TB in Brazil, reported between 2003 and 2008, including those whose test was requested but not recorded and those in whom a test was not done.

## Methods

### Ethics statement

The databases were obtained under the rules for release of the Secretariat of Health Surveillance and Health Care Department of the Ministry of Health, ensuring the confidentiality and non-disclosure of individual identifiers. The study was approved by the Brazilian National Commission for Research Ethics and by the International Union Against Tuberculosis and Lung Disease's Ethics Advisory Group (EAG).

### Design

This was a retrospective cohort study consisting of record reviews of data from the national TB reporting system (*SINAN – Sistema Nacional de Agravo de Notificação*).

### Setting

The study included six States of Brazil selected because they include a large percentage of TB cases (37% in 2008) in the country and they have a higher level (top quintile) of completeness of the data on HIV status for TB cases in the national reporting system. Between the beginning of the AIDS epidemic in Brazil and 2009, these States also represented 62% of the AIDS cases reported in the country [9]. States included were: Santa Catarina, São Paulo, Rio Grande do Sul, Espirito Santo, Mato Grosso do Sul and Paraná. The standard TB treatment for new cases during the study period was: 2 months of isoniazid, rifampicin and pyrazinamide followed by 4 months of isoniazid and rifampicin (2 HRZ/4 HR). In Brazil, anti-TB drugs are only provided by the public health system.

### Patient Sample/Study Population

The study population included all new TB cases in the six selected States in Brazil in the period from January 1, 2003 to December 31, 2008, i.e., a total of 161,481 cases. The six-year study period was chosen in order to evaluate a substantial number of new TB cases and this represented a period during which the Brazilian government declared TB to be a national public health priority. The relatively long time span avoided potential short term fluctuations in case reporting and was terminated in 2008 because this was the most recent year for which final and consistent data were available in SINAN.

### Variables, data collection

A new case was defined as a TB case who was never treated with anti-TB drugs for as much as one month; or those who had completed previous TB treatment over 5 years ago. We excluded those classified as retreatment cases, defined (in that time period, 2003 to 2008) as those who presented as a TB case and had completed treatment less than 5 years ago [10].

A TB case was defined as a patient presenting one or two positive sputum smears and/or a positive culture result. However, if bacteriological exams were negative, one could be reported as a TB case based on symptoms (such as cough for over 3 weeks, fever, night sweats and weight loss), epidemiological history (having had contact with a known TB patient) and complementary exams suggesting the disease (chest X ray, tomography, tuberculin skin test and histopathology exams) [10].

A patient was considered HIV infected when a screening HIV test by Enzyme-Linked Immunoabsorbent Assay (ELISA) was positive, followed by a confirmatory positive test. The confirmation should be obtained by a second blood sample when a new ELISA and an indirect immunofluorescence test or a western blot were positive [11].HIV status was defined as positive, negative (based on a recorded result in SINAN); HIV test requested but no result recorded; or, HIV test not done. Information on HIV status should be informed by the time the case gets reported, and it can be updated during the course of treatment. The National Tuberculosis Program (NTP) recommends that the epidemiological surveillance services send requests to health services during the course of treatment if they receive case notifications with the HIV field either empty, or with no result. Cure was defined as completed treatment with 2 negative smear microscopy examinations (one during the course of treatment and one at the end) for the pulmonary cases initially smear positive; or, based on clinical, radiological and complementary examination criteria in those who did not produce sputum for a smear examination. The latter criterion was also applied for the extra-pulmonary cases (clinical cure not bacteriologically confirmed).

A patient was defined as a defaulter if he or she did not come to the health service for more than 30 days after the expected date of consultation, and in the case of directly observed therapy (DOT), the 30 day period started on the last day of observed drug intake.

Death was considered as the outcome when there was knowledge of the patient's death during the course of treatment, regardless of cause of death. Multidrug-resistant tuberculosis (MDR-TB) was defined as a case that was documented as resistant to at least rifampicin and isoniazid after drug susceptibility testing.

All variables collected and used in this analysis came from the informatics TB reporting system. Data were abstracted electronically by Ministry of Health staff at central level, where information flows from the lower levels (municipal and state) and gets consolidated. Variables included sex, age, education, type of TB, thoracic X ray reading, HIV status and TB treatment outcomes. Data were abstracted between February and May of 2011.

### Data Analysis and Statistics

Data were tabulated and proportions compared for categorical variables using the Chi-square test. All the variables, considering the number of missing data was not too substantial, such as schooling and race, were examined for their association with the outcome (to evaluate their potential as a predictor) and also exposure (to identify potential confounders) for inclusion in the multivariate models. Relative risks (RR) and relative risk ratios with 95% confidence intervals (CI) were calculated using log binomial [12] and multinomial logit models to determine relationships using the softwares Stata™ version 7 and R version 2.12.0.

In bivariate analysis, we investigated the potential for confounding and other associations between potential explanatory variables and treatment outcomes. Any such variable was included in the models, along with the state of residence. As São Paulo State accounted for a high proportion of cases in the dataset, we determined whether adjusting for this variable affected the detected differences and associations.

In order to analyze the treatment outcome patterns, we had two different approaches. The first one compared cure rates against all other outcomes, defined as unfavorable. The second one looked at default and death separately, in relation to those who were cured.

The second model considered three levels of the outcome (cure, default and death), as opposed to the first one when we looked at cure versus all other outcomes, considered unfavorable. This way we were also able to examine the effect of HIV status separately on default and death. We included type of TB and age in the model, as per the strategy defined above. In addition, an interaction term between type of TB and HIV status was tested. Relative risk ratios were obtained comparing HIV positives to negatives, and the result was the factor by which the relative risk for default compared to cure was expected to change, given that the other variables in the model were held constant. The same procedure was carried out for death compared to cure.

### Sample size considerations

All patients meeting the inclusion criteria (new TB cases) in the study period in the selected States were included in the study.

## Results

A total of 161,481 new TB cases were reported to SINAN between 2003 and 2008 in the selected States. Basic demographic and clinical characteristics of the cases are reported in table 1. A majority of cases (67%) were males, almost 40% had less than 8 years of education and almost 80% had pulmonary TB. A high proportion of the cases were reported in the State of São Paulo (59%). Of the total study population, 68% (109,820) was known to have been HIV tested, of whom 20,881 were HIV-positive (19% of those tested) and 88,939 were HIV-negative. Of those with no HIV result, 10,567 had a test requested but no result was available and in the remainder the HIV test was not done. Seventy-eight percent of all cases had a smear microscopy exam done, and 70.6% of those had a positive result. As far as culture, 19.7% had a result for it, and 65% of those were positive.

**Table 1:** Characteristics of new tuberculosis cases in 6 States of Brazil between the years of 2003 and 2008 (n = 161481).+

	N (%)
Age (median, IQR)	37 (26–49)
Sex	
Male	108151 (67)
Female	53322 (33)
Education (years of study)	
0 to 4	22076 (13.7)
5 to 8	42152 (26.1)
9 to 11	21989 (13.6)
University degree	8458 (5.2)
Missing	66806 (41.4)
Race	
White	54433 (62)
Black	9531 (10.9)
Asian	725 (0.8)
Mixed (mulatto)	14492 (16.5)
Indigenous	1224 (1.4)
Not provided	7306 (8.3)
Type of TB	
Pulmonary	127260 (78.8)
Extra pulmonary	27654 (17.1)
Pulmonary and extrapulmonary	6539 (4)
Thoracic X ray	
Suspect	126875 (82.6)
Normal	10354 (6.7)
Other pathology	2162 (1.4)
Not done	14159 (9.2)

+ Data are presented as actual numbers and proportions for categorical variables, and median (and interquartile ranges) for continuous variables.

TB treatment outcomes were best for those who were known to be HIV negative and worst for those known to be HIV positive (see table 2). TB treatment outcomes for those in whom an HIV test was requested but the result was not available were better than those in whom the test was not done.

**Table 2:** TB treatment outcomes by HIV status in new TB cases diagnosed in 6 States of Brazil between 2003 and 2008.

TB treatment outcomes											
HIV Status	Total (N)	Cure (N, %)		Default (N, %)		Death (N, %)		Transfer (N, %)		MDR-TB (N, %)	
Negative	87693	75185	(85.7)	6185	(7.0)	3652	(4.2)	2608	(3.0)	63	(0.07)
Positive	20426	11369	(55.7)	2792	(13.7)	4705	(23.0)	1536	(7.5)	24	(0.12)
Test requested but no result recorded	10035	7568	(75.4)	1166	(11.6)	736	(7.3)	563	(5.6)	2	(0.02)
Test not done	40043	27953	(69.8)	4570	(11.4)	5016	(12.5)	2478	(6.2)	26	(0.06)
Total	158197	122075	(77.2)	14713	(9.3)	14109	(8.9)	7185	(4.5)	115	(0.07)

In bivariate analyses, in reference to the HIV negative group, patients with a positive HIV-status or unknown HIV status had a higher probability of having an adverse treatment outcome (risk ratios ranged from 1.7 to 3.1, all p-values<0.05).

Type of TB was also associated with HIV status. Those who were HIV positive had a higher risk of presenting extrapulmonary and disseminated forms than the pulmonary form, when compared to HIV negative patients (RR 2.08, 95% CI 2.02–2.13; and 3.15, 95% CI 3.05–3.26 respectively – see table 3).

**Table 3:** Form of TB presentation by HIV status among new TB cases diagnosed in selected States of Brazil between 2003 and 2008 (n = 109815).

HIV status	Form of TB			Total (N, %)
	Pulmonar (N, %)	Extrapulmonar (N, %)	Disseminated (N, %)	
HIV negative	72216 (81.2)	13915 (15.6)	2805 (3.2)	88936 (100)
HIV positive	12390 (59.3)	6078 (29.1)	2411 (11.6)	20879 (100)

In the first multivariate model, HIV positive TB cases were 3 times more likely (adjusted risk ratio 3.09, 95% CI 3.02–3.16) to have an unfavorable outcome, when compared to HIV negative TB patients. Risk ratios for patients with no HIV test results confirmed the associations already shown in table 2. The risk ratios of an unfavorable outcome for the groups with HIV test requested but with no result recorded, and those without an HIV test, again using the HIV negative group as the reference, were 1.71 (95% CI 1.64–1.78) and 2.09 (95% CI 2.05–2.14) respectively.

The inclusion of the variable related to state of residence did not significantly change the point estimates for the risk ratios derived from the model or the direction of the observed association.

**Figure 1:**
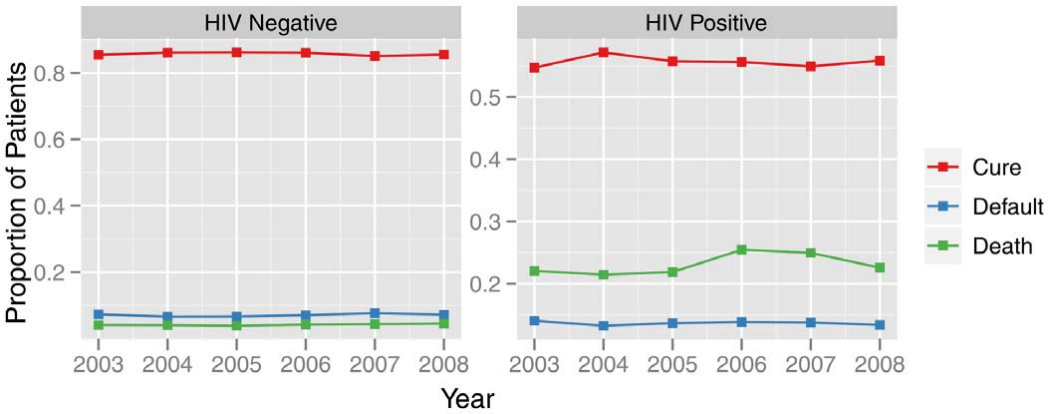
Treament outcomes (cure, default and death) for new TB cases by HIV status, Brazil 2003–2008. This figure shows the proportion of new TB cases and their outcomes, by year, during the study period. It demonstrates that these proportions remain stable, at different levels for each of the outcomes, over the 6-year period, for both HIV positive and HIV negative patients. It also highlights the differences by HIV status, especially regarding cure and death rates.

The proportion of patients with known HIV status increased overtime, from 62% in 2003 to 74% in 2008, whereas the group without an HIV test done decreased from 31% to 21% by the end of the study period. Nevertheless, the cure, default and death rates for both HIV positive and negative groups remained stable overtime (figure 1).

In the second multivariate model, using cure as the reference group, death was shown to get an increase in RR by a factor of 9.24 with HIV positivity (95% CI 8.78–9.72), whereas this factor is 2.88 (95% CI 2.74–3.03) when we looked at default. In other words, if a patient is HIV positive, the relative risk for default compared to cure, and death compared to cure, would be expected to increase by a factor of 2.88 and 9.24 respectively, given the other variables in the model are held constant. The interaction term between type of TB and HIV status proved to have no significant effect on the model estimates.

## Discussion

Our study showed a striking difference in TB treatment outcomes between those known to be HIV positive and those known to be HIV negative with intermediate levels of treatment success in those with unknown HIV status. Moreover, a substantial proportion of TB cases did not have an HIV test result recorded (either not recorded in the system or not done), which falls short of recommended levels for HIV testing.

The fact that cure, default and death rates for both HIV positive and negative groups remained stable overtime deserves attention. These results indicate little progress in the implementation of TB/HIV collaborative activities, and that only the offer of HIV testing for TB patients is not sufficient for therapeutic success, and reinforces the need to ensure the provision of comprehensive care to co-infected patients. In addition, the national guidelines for TB/HIV case management during the study period [10] remained unchanged, and recommended to prioritize TB treatment, and only after clinical improvement the patient was evaluated for eligibility for antiretroviral therapy (ART), by performing CD4 cell count and viral load tests. This approach caused delays in initiating ART in TB patients who, by current guidelines, would be eligible for it [13].

The TB treatment outcomes in those known to be HIV positive were relatively poor, with low cure rates and high death rates. A recent study compiled results from several other studies on the effect of HIV co-infection on the outcome of TB treatment [14]. These studies were smaller, not population based and used regimens slightly different than that used in Brazil during the study period, and the cure rates observed ranged from 57% to 91%. Of note, in 4 of these studies the patients were not on antiretroviral treatment (ART) and the cure rate was close to 80%. We have no information about ART usage in the patients in our study, but even assuming a worst case scenario where no patient was placed on ART, the observed outcome was not very satisfactory. Death rates in these smaller studies were also high, but in some of these studies follow-up continued beyond the end of anti-TB treatment, while in our study we assessed treatment outcomes only during the course of anti-TB treatment.

The intermediate treatment success noted in those in whom the HIV test result was unknown (either not recorded or not done) suggests that this group of patients contained a proportion who were HIV positive who might have benefited from integrated HIV and TB care, if their results had been known and high quality of HIV care offered to them.

The States included in this study are considered to be States with a better than average health care system, especially São Paulo and the three States from the Southern Region (Rio Grande do Sul, Santa Catarina and Paraná). These are not considered as representative of the country as a whole. Despite this, the cure rate for new TB cases that were known HIV positive at 55% was low compared with that observed in patients who were HIV-negative. We do not know the reasons for this as there were no data available in the TB national system on modalities of HIV care and ART usage. Although this is a limitation of our study, it points to the need for future research that can potentially be carried out by linkage of national TB and HIV/AIDS programs databases on a national level. Work has previously been carried out in Brazil on the effect of ART on outcomes of people living with HIV and the effect of ART on TB incidence [15], [16] and on factors associated with co-infection itself [17], [18]. However, less attention has been paid to the effect of HIV status on TB treatment outcomes. Two cohort studies [19], [20] looked at the influence of HIV infection on mortality of patients undergoing TB treatment in Rio de Janeiro, and their findings confirm the increased mortality risk for HIV positive individuals not on ART as compared to HIV negative patients. In addition, one of them showed an excess risk, although not statistically significant, of mortality when comparing HIV positive patients on ART to HIV negative patients [20]. These findings are consistent with our results. A possible explanation, confirmed in these two cohort studies [19], [20], is the higher clinical severity of disease at presentation. In our study, we also showed that those with worse outcomes, including increased mortality, were the HIV positive individuals, and these were more likely to present extrapulmonary and disseminated forms of TB. The level of immunosuppression at the time of TB diagnosis among those HIV infected can be a consequence of missed opportunities by the health care system to provide the diagnosis or of delays in the patient seeking care to avoid receiving a positive HIV result [18].

Overall the group of patients without an HIV test done had worse treatment outcomes than the group in which a test was requested but no result was recorded in the public health system. This observation is not easily explained and warrants an in depth investigation of the profile of each group, ideally by obtaining the actual HIV status of these patients or gathering information on whether the test was carried out or not. This analysis is beyond the scope of this paper.

Another important point that came out of our study was the treatment outcome of patients without a known HIV status. Regardless of whether the test was requested or not, the outcomes were at an intermediate level between the HIV positive and negative groups. This indicates that there were probably HIV infected TB patients among this group, resulting in their treatment outcomes being different from the HIV negative group. There was no difference in the distribution of any other variables when these groups were compared. Therefore, the unknown variable, i.e., HIV status, might be responsible for the observed pattern. This finding has important policy and programmatic implications. The offering of HIV testing for new TB cases is supposed to be universal, but our finding demonstrates that this does not occur. It is of concern that this situation was detected in States with a fairly good health services organization and some degree of integration between TB and HIV Programs. Of the 6 states participating in the study only Sao Paulo published a decree in 1998 [21], which recommends the provision of anti HIV testing for all TB patients and also recommends the establishment of a flow between the TB and HIV/AIDS health units for the comprehensive care of co-infected patients. During the study period we did not observe any additional actions to increase HIV testing in TB services beyond the national recommendations. Moreover, HIV testing for TB patients was not performed in the TB clinic, and therefore had to rely on the willingness of the individual to seek testing elsewhere (laboratory or center for anonymous HIV testing). Obviously, there is a long way to go to ensure that all TB cases are tested for HIV, and as important as the testing itself, that this information makes its way into the national reporting system.

To improve the outcomes mentioned above, the National HIV/AIDS Program officially recommended rapid testing for HIV diagnosis in 2009, making HIV testing available at TB services [22]. In 2010, both TB and HIV/AIDS Programs issued guidelines defining the HIV/AIDS services as the reference for treatment of co-infected patients [23]. The expected consequence of such strategies will be an increase in the proportion of TB cases tested for HIV and an improvement of success rates of TB treatment in TB/HIV co-infected cases. Future studies will allow us to confirm if these policies, not present during our study period, had the expected result.

Our study is a very large study and evaluates a substantial proportion of the population of Brazil. It strongly confirms that the access to HIV testing is not as high as it should be, even though the States studied would have been expected to perform well in this regard. It is possible that these results reflect the situation in many similar countries with a high TB and HIV burden.

One limitation of our study is that we did not have access to ART history among our group of HIV co-infected patients. It is likely though that most of them were not receiving ART and care or were diagnosed late in the course of their HIV disease. The large proportion of deaths in this group reinforces this hypothesis. Another limitation is that the study covered only some of the States in the country. Hence, it cannot be interpreted as representative of the whole country. In addition, since we were dealing with patient-level data, we could not examine health system related variables, such as quality of the care provided by the facility, coverage of the Family Health Programs and DOTS strategy in the area where the patient lived. Nevertheless, since we intentionally chose States with good completeness of data, which in turn is a reasonable marker for a well-organized service, it is of concern that the situation we assessed is far from optimal.

Information on DOT could not be included in the analysis due to the limited availability of information on this variable in the database. Until 2007, the only field in the TB case report form related to DOT indicated that it was recommended for that patient, but there was no confirmation that it was actually carried out throughout the treatment. During the study period, the number of drug intake events that should be observed to consider that patient as having had DOT completed was defined as three weekly observations during the intensive phase and one weekly during the continuation phase [10]. In 2010 the NTP released its revised National Guidelines [23], stating that DOT is defined as at least 3 weekly observations both during the intensive and continuation phases of treatment. This definition has been also refined stating that the choice and strategy for DOT needs to be defined taking into consideration the patient, health staff and the structure of the health service responsible for that individual's treatment. Data from the NTP indicate that in 2009 nationally, the cure rate for those TB cases on DOT was 77.3%, compared to 68.7% for those patients not on DOT. The latter group had also higher default (11.2%) than the former (7.1%) [3]. It will be of interest to repeat this analysis in the future using data from a period when the revised definition of DOT will be fully implemented throughout the country.

The results of our study indicate the need to urgently implement the TB/HIV collaborative activities, which in turn will improve the care of co-infected patients. More patients need to be HIV-tested. It is also essential to evaluate the quality of care of those known to be HIV positive to determine if the unfavorable outcomes of treatment can be improved through better HIV care and treatment, if so to extend this care to all those who are HIV positive.

### Conclusion

Care for HIV infected new TB cases needs to be closely monitored by health authorities in Brazil to guarantee that this group accesses the benefits that therapy can bring. In addition, HIV testing needs to become effectively universal for new TB cases, so that the care mentioned above can be provided in a comprehensive and timely manner. Finally, it is likely that a certain proportion of TB patients are HIV-tested but the results are not being documented in the routine information systems, which points out to the need for more training and awareness from all health professionals to properly document these actions. This is the only way to generate the solid evidence policy makers need to make decisions that will improve health outcomes for the populations that need care.
